# Ontario Veterinary College

**Published:** 1887-01

**Authors:** 


### ONTARIO VETERINARY COLLEGE.

The Ontario Veterinary College opened in the large lecture-room of the college in Temperance Hall. There were between two and three hundred students present, and on the entrance of Mr. Andrew Smith, V.S., President of the College, all rose to their feet and cheered him enthusiastically. The class assembled to listen to the introductory lecture of the session, was one of which the college may well feel proud. The students were clever, intelligent looking men, apparently ranging from twenty to thirty-five. A majority are farmers' sons, who have already more or less practical knowledge of the handling and general characteristics of domestic animals; but whether farmers' sons or not. all looked and acted as though they had come for study and hard work, rather than the mere putting in of the time.

Dr. Smith, on taking the platform, after the storm of applause which greeted his appearance had subsided, expressed his satisfaction at meeting his old students of last year, as well as the large class of freshmen which he saw before him. In his address, which occupied about three-quarters of an hour, he gave an interesting sketch of the rise and progress of veterinary science, from its inception among the Egyptians to the present day. That portion of the address which dealt with the history of the profession in France, England, Scotland, the United States, and Canada, was particularly interesting and instructive, and was listened to with the closest attention by all present. In conclusion he pointed out in glowing terms the bright prospects which were open to the diligent and persevering veterinarian on this continent, where the field for practice was almost unlimited, only partially developed, and as yet comparatively unoccupied. He also dwelt with some warmth upon the manner in which the graduates of Ontario Veterinary College had distinguished themselves in the United States and elsewhere, both in practice and veterinary literature.

The Christmas examinations of the Ontario Veterinary College were held on December 20th. The following gentlemen, third year students, passed the official Board of Examiners, and were awarded the Diplomas of the Agricultural and Arts Association :—

Geo. Dunn, Simcoe, Ont.; Jacob McFetzer, Center Valley, Pa., U. S.; John F. Fisher, Brandon, Manitoba; D. Bell, Brampton, Ont.; William Hunter, Brampton, Ont.; W. S. Henderson, Arthur, Ont.; J. H. Hennessey, Hamilton, Ont.: Wm. S. Hibbard, Caledonia, Ont.; George N. O'Leary, Pickering, Ont.; R. H. McIninch, Dakota, U. S.; W. H. McNaughton, Vienna; Ohio, U. S.; J. E. Rayen, Girard, Ohio, U. S.; W. H. Riddell, Orangeville, Ont.; C. Wagg, Goodwood, Ont.; A. C. Wolfe, Goodwood, Ont,; J. H. Thornton, Georgetown, Ont.; M. E. Young, Belville, Ohio, U. S.; T. H. Sterling, New Hamburg, Ont.

J. E. Rayen, J. F. Fisher, W. S. Henderson, and G. N. O'Leary passed with great credit.

C. H. Shirland, Madrid, N. Y., passed a primary examination in Materia Medica.

